# Exohedral Functionalization of Fullerene by Substituents Controlling of Molecular Organization for Spontaneous C_60_ Dimerization in Liquid Crystal Solutions and in a Bulk Controlled by a Potential

**DOI:** 10.3390/polym13162816

**Published:** 2021-08-22

**Authors:** Malgorzata Czichy, Alessia Colombo, Pawel Wagner, Patryk Janasik, Claudia Dragonetti, Rathinam Raja, David L. Officer, Leeyih Wang

**Affiliations:** 1 Faculty of Chemistry, Silesian University of Technology, Strzody 9, 44-100 Gliwice, Poland; Patryk.Janasik@polsl.pl; 2 Department of Chemistry, University of Milan, UdR dell’INSTM, Via Golgi 19, 20133 Milan, Italy; alessia.colombo@unimi.it (A.C.); claudia.dragonetti@unimi.it (C.D.); 3 ARC Centre of Excellence for Electromaterials Science, Intelligent Polymer Research Institute, University of Wollongong, Northfields Avenue, Wollongong, NSW 2522, Australia; pawel@uow.edu.au (P.W.); davido@uow.edu.au (D.L.O.); 4 Center for Condensed Matter Sciences, National Taiwan University, Taipei 10617, Taiwan; rathinamraja@ntu.edu.tw (R.R.); leewang@ntu.edu.tw (L.W.); 5 Institute of Polymer Science and Engineering, National Taiwan University, Taipei 10617, Taiwan

**Keywords:** fullerenes, C_60_ dyads, C_120_ dimer, π-dimerization, liquid crystals

## Abstract

A study was carried out on the possibility of orderly and spontaneous dimerization at room temperature of C_60_ cages in fullerene liquid crystal fullerene dyads (R-C_60_). For this purpose, dyads with a structural elements feature supporting π-stacking and Van der Waals interactions were tested, due to the presence of terthiophene donors linked through an α-position or dodecyloxy chains. In addition, this possibility was also tested and compared to dyads with shorter substituents and the pristine C_60_. Research has shown that only in dyads with the features of liquid crystals, π-dimerization of C_60_ units occurs, which was verified by electrochemical and spectroelectrochemical (ESR) measurements. Cyclic voltammetry and differential voltammetry studies reveal π-dimerization in liquid crystal dyad solution even without the possibility of previous polymerization (cathodic or anodic) under conditions in the absence of irradiation and without the availability of reaction initiators, and even with the use of preliminary homogenization. These dyads undergo six sequential, one-electron reductions of π-dimer (R-C_60_···C_60_-R), where two electrons are added successively to each of the two fullerene cages and first form two radical anion system (R-C_60_)^•−^(R-C_60_)^•−^ without pairing with the characteristics of two doublets. Similarly, the second reductions of π-dimer occur at potentials that are close to the reduction potential for the conversion to a system of two triplet dianions (R-C_60_)^2−^(R-C_60_)^2−^. Electron paramagnetic resonance spectra indicate a significant interaction between C_60_ cages. Interestingly, the strength of intermolecular bonds is so significant that it can overcome Coulombic repulsion, even with such highly charged particles as dianions and trianions. Such behavior has been revealed and studied so far only in covalently bonded C_60_ dimers.

## 1. Introduction

**Figure 1:**
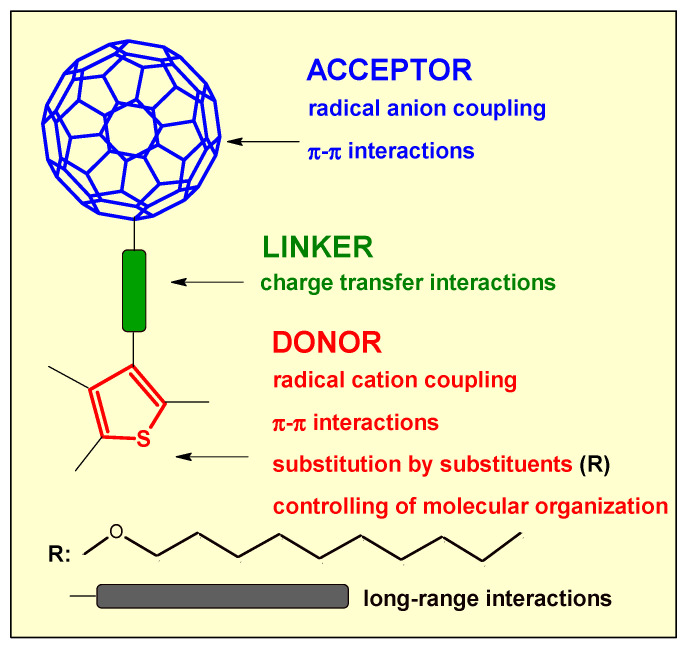
Structural fragments of C_60_ dyads vs. properties tested in this work.

The formation process and properties of fullerene polymers are important for their potential applications in optoelectronics and nanotechnology applications. In the pristine solid state, the fullerene molecules are bound together by a weak, van der Waals (vdW) interaction (Figure 1). However, strong inter-fullerene bonds have been observed under: high temperatures [1], electron-beam [2], irradiation [3], and high-speed vibration milling [4], during their fusion inside carbon nanotubes, while subjected to effective high-pressure conditions [5], charge-transfer polymerization mediated by metals [6,7,8,9], or plasma-induced polymerization [10]. The predominant inter-fullerene bonding for the most studied pristine C_60_ motifs are four-membered carbon rings arising from [2 + 2] cycloaddition reactions [11] or single C-C covalent bonds bridging to dimeric form and then [12], usually, propagating to one- (1D) [13] and two-dimensional polymers (2D) [14]. In turn, the interaction between such low-dimensional polymers in 3D assemblies is generally weak, which reduces the formation of covalent bonds [15]. On the other hand, the targeting of directional non-covalent forces, such as aromatic interactions, hydrogen bonding, and dipole–dipole interaction could enable control of the self-assembly process for creating more ordered fullerene structures. Fullerene-building materials are yet to become essential components of practical electrical conductors due to high control over the material’s morphology at the stability of the nanoscale [16]. Therefore, the study of the covalent assembly process and long-range interactions is important and can also be investigated by the electrochemical methods.

The fullerene dimers are essential subunits in the formation of fullerene polymers. Therefore, they are also the subject of our research. Recently, much attention has been paid to the characterization of C_60_ dimers: [2 + 2] dumbbell-shaped C_120_ [17], C_120_ with two intact C_60_ cages joined by six C–C single bonds [18], single and doubly bonded C_120_^2−^ dimers in the ionic complex with manganese(II) tetraphenylporphyrin [19], C_120_O [20], and C_120_O_2_ obtained under oxygen condition [21].

The stability of these dimers depends on the binding strength provided by intramolecular interactions and distances between the C_60_ molecules. The reactivity and electronic properties of C_60_ depend heavily on non-covalent intermolecular interactions, which result in specific molecular arrangements and crystal packing modes, and morphology also depending on the phase. The interaction between fullerene molecules in the solid film is not significant and very small. Above 300 K, the C_60_ molecules spin freely about their long axes, which are aligned parallel to one another, and at higher temperatures (573 K), the molecules are then found to spin or tumble around their lattice positions, about randomly-oriented axes [22]. The distance between the center-of-mass positions in the face-centered-cubic (fcc) structure of the two nearest-neighbor C_60_ molecules is 10.01–10.42 Å to 130 K in solid C_60_ [23]. Above T 260 K, both weak intermolecular interaction and the spherical shape allow the molecules to spin rapidly about their lattice positions, which also inhibits the coupling processes [24]. In confined C_60_, the nearest-neighbor cage distance approaches a value of 8.45 Å near 10 GPa [25], so the spontaneous dimerization and polymerization of C_60_ is unlikely to take place without any external stimuli. New phases appear under applying high pressure and temperature in which the equilibrium distance between nearest neighbor C_60_ molecules gets shortened from about 10 Å to 9.0 Å in the crystalline state [26]. Dimerization by radicals coupling mainly occurs when radicals are located primarily on the two separate C_60_ cages and the most obvious means of communication would come about between π-π overlap in the region where the two cages are closest to. Reducing the distance between C_60_ cages also occurs by selecting the donor and acceptor in the fullerene complex. Neutral and negatively charged fullerenes (C_60_, C_70_) form a large variety of donor–acceptor complexes with different classes of organic and organometallic compounds e.g., metalloporphyrins [19] or metal phthalocyanines [27], amines [28] or solvated metal cations [29]. Neutral or negatively charged fullerenes form complexes with nitrogen-containing ligands, cations, and some anionic ligands as coordinated, self-assembled crystalline structures. The structure of solid donor–acceptor complex has often been established using single-crystal X-ray analysis [30,31], so determining the pattern in liquid-crystalline solutions is already more of a challenge. 

Exohedral functionalization of C_60_ can activate the bridgehead positions owing to steric and π-electron density polarization effects. Hence, dimers of exohedral fullerenes offer a model system for investigating the interaction between electron spins, and the stability of the radical species strongly depends on the nature and quantity of substitution [32,33,34,35,36]. Various dyads, especially monosubstituted fullerene (C_60_-R) and their radicals, have been researched. Sterically demanding groups, such as the *t*-butyl group, give rise to rather tenacious radicals [37]. Monoalkyl radical adducts show considerably hindered rotation around the C_60_–R bond [38,39,40]. Fullerene radicals with alkyl or hydrogen substitutions can dimerize to form singly-bonded 4,4′-disubstituted adduct [41]. The electrochemistry of fullerene and fullerene dimers is of particular interest, as the redox behavior of dimeric fullerenes reflects the electronic interaction between the fullerene cages [42,43,44]. Potential values are essential for understanding and predicting the homogeneous redox properties of each particular fullerene derivative, as well as the areas of their possible application.

Substituted C_60_-derivatives, fullerene homopolymers, and the elongate-shaped moiety e.g., C_70_ lead to a more complicated rotational behavior than observed in the solid state. Most of the exohedral derivatives are also dissolved in non-aromatic solvents, where interesting effects can be observed, not only related to the functionalization itself, but also to a change in the type of intermolecular interactions, which can favor a specific organization, a decrease of intermolecular distance, and promote the coupling process. As an analogy can be point out the increasing external pressure, which induced more efficient dimerization of C_60_ to produce C_120_ in a co-crystallite than in the amorphous bulk [45]. Co-crystallizations and supramolecular assemblies are giving rise to an explosion of new fullerene-containing liquid crystals (LC), functionalized often by different mesogenic units: hexaalkyloxytriphenylene [46,47], pyrrolidine [48,49,50], triethyleneglycol chains [51], cyanobiphenyl [52], alkoxy chains [53], and others [54]. Liquid crystalline fullerenes can be used in optical and electronic applications; recently, the interest has turned to formation strategies, where they are assembled spontaneously into macroscopic rods [55,56], vesicles [57], bent-cores [49], and discs [58]. Self-assembling of fullerenopyrrolidine with long alkyl substituents occurrs in solvents of different polarities to form ordered supramolecular ensembles with the 1D, 2D, or 3D architecture such as vesicles, disks and cones, containing bilayers as the fundamental structural unit. The lamellar structure can be formed by the crystal and the liquid crystal, where the distance between the fullerene layers can be equal to around 22 Å, while the short distances to 9.80 and 10.16 Å between neighboring fullerene cores suggest a strong C_60_–C_60_ interaction within the same layer. Further, the introduction of alkane or π-additives can break the balance between the vdW and π–π interactions in liquid molecules, allowing additive-directed conversion from a liquid to a highly ordered LC or a gel. For example, dimerization was observed in crystals: bithiophene-fulleropyrrolidine with ethoxy linker [59], C_60_ derivatives containing mono-, bis- and trishexadecyloxyl chains [60]. The supramolecular organization of such fullerene derivatives was largely associated with π-π interactions between fullerene fragments and vdW interactions between asymmetrically arranged alkyl chains. Such compounds are promising as capacitors in which the fullerene fragments constitute conducting layers divided by an insulating layer formed by alkyl groups [61]. However, the development of fullerene-based devices very often requires dedicated engineering of the molecules, that is, functionalization of the molecules for controlling their self-organization, which can also be indirectly tested by electrochemical techniques.

**Figure 2:**
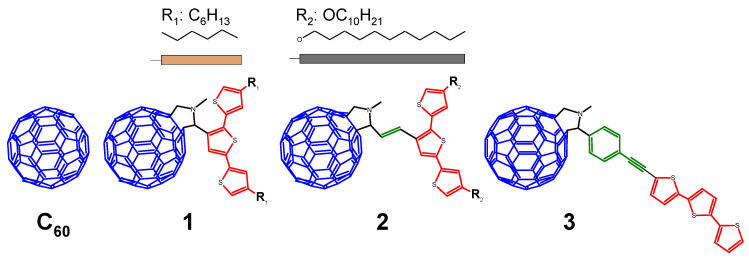
The structure of the studied C_60_ dyads—**1**, **2**, and **3** and pristine **C_60_** fullerene.

Thus, fullerene dimerization can be a result of the formation of self-assembling ionic complexes dyad and/or charge transfer (CT) in donor–acceptor complexes, but not mentioned for liquid-crystalline fullerene solutions. We want to use the possibility of manipulating the weak forces in C_60_ fullerene liquid crystals to investigate the electroreduction, because different distances can be observed between C_60_ cages, depending on the hierarchical structure. We are focusing on the indication that the appropriate modification can cause a specific electrical interaction and dimerization process between C_60_ cages in three derivatives (**1**, **2**, **3**, Figure 1) in solid and liquid states. A preliminary electrochemical study of **2** derivative with long decyloxy chains (R_2_) was reported in our previous paper [62]. There, an interesting behavior of the reduction process was observed, which was initially indicated on the coupling process of radical anions, which was initially assigned. However, the detailed evolution of voltammograms during electroreduction of **2** in the solution and solid state and after polymerization of the terthiophene segment is not clarified. This derivative is characterized by a donor and acceptor connected with a push-pull vinyl linker, which results in the polarization of the whole molecule and also distances individual segments from each other. For comparison, a derivative **1** containing shorter hexyl substituents (R_1_) and terthiophene segment directly substituted to C_60_-pyrrolidine was also investigated. The vdW interactions between alkyl and alkoxy chains, but also π-π stacking interactions between p_z_ orbitals of the thiophene rings, play a role in aggregation phenomena, and these interactions arbitrate liquid and solid-state crystal formation. Therefore, we also tested the electroreduction behavior of the last derivative (**3**)-α-substituted terthiophene linked to C_60_-pyrrolidine via insulating the phenylacetylene segment. In the case of this last dyad, it was assumed that π-conjugation was broken between the terthiophene and fullerene, and instead, π-π stacking was increased between terthiophenes of two dyads. Additionally, the C_60_ reduction processes in pendant-type polymers obtained after electrochemical oxidation of terthiophene fragments were also studied. The reference sample was pristine fullerene (**C_60_**) (Figure 2).

Therefore, our research focused on the structure of fullerene dyads; the possibility of charge transfer; specific packing in self-assembled structures; decreasing the distance, especially between the charged fullerene cages in liquid-crystalline solutions; and the possibility of interaction with another component of the solution, with electrolytes necessarily present in an electrochemical measurement.

## 2. Materials and Methods

### 2.1. Materials

C_60_ (99.5% Sigma-Aldrich, sp. z o.o., Poznan, Poland); tetraethylammonium tetrafluoroborate (E_1_, 99%, Sigma-Aldrich); tetrabutylammonium tetrafluoroborate (E_2_, 99%, Sigma-Aldrich) dried under a vacuum; dichloromethane (DCM, HPLC, 99.8%, Sigma-Aldrich); acetonitrile (ACN, Sigma-Aldrich, 99%); Ru_3_(CO)_12_ (Sigma-Aldrich); ferrocene (Fc, 98%, Sigma-Aldrich); and argon (6.0, SIAD Group, Ruda Śląska, Poland).

### 2.2. Synthesis

The synthetic procedure, the yield of this process, and confirmation of the structure of each sample (SYNAPT XS High Resolution Mass Spectrometer, 1H- and 13C-NMR spectroscopy—Brucker Advance 400, Billerica, MA, USA) were presented in our previous papers: **1** [63], **2** [47,62], **3** [42].

### 2.3. Electrochemical and Spectroelectrochemical Measurements

Cyclic voltammograms were obtained using a Bio-Logic SP-150 and CH Instruments Electrochemical Analyzer model 620. A single-cell and three-electrode setup was used (eDAQ Pty Ltd., Denniston East, Australia): 2 mm^2^ of platinum disk (working electrode), platinum coil (counter electrode), and Ag|Ag+ pseudo-reference electrode calibrated with ferrocene as the internal standard. The sample concentration was 1.0 mM (in the presence of 0.1 M E_1_ or E_2_) in DCM. Polymer films were prepared on platinum electrode by electrooxidation after the first oxidation peak. The potential was changed at a rate of 50 mV/s. Argon was bubbled before electrooxidation and electroreduction. ESR spectroelectrochemical measurements were acquired using a JEOL JES FA-200 X-band spectrometer with the following parameters: 0.6 mT (modulation width); 1 mW (microwave power); and 200 (amplitude). A capillary quartz spectroelectrochemical cell was used, equipped with a Pt wire working electrode in monomer solution (1 mM) or with a polymer coating, Ag wire pseudoreference electrode (potential calibrated versus standard potential of Fc|Fc^+^ couple) and Pt coil as a counter electrode. The surface imaging of electrochemically obtained polymers was performed with Leica DM 2500 B microscope with 50 lenses was used.

### 2.4. Theoretical Calculations

DFT calculations were performed in Gaussian 09 [64] software installed on PL-Grid Infrastructure. Input files and molecular orbital charts were prepared using Gabedit 2.4.7 software [65]. It was decided to use two functionals for the calculations. Functional ωB97X-D combined with 6-31G(d,p) basis set was used to calculate the geometries of monomers and fullerene dimers, which were presented in this work—this functional is recommended for calculations taking into account non-covalent interactions such as π-π stacking or hydrogen bonds [66,67]. Next, frequency calculations were systematically achieved (at the same level of theory) to confirm the minimum nature of the optimized geometries. Then, the functional B3LYP was used to calculate the single point to obtain the energy and localization of the electron orbitals. B3LYP/6-31G(d,p) was used due to some reasons: the shape and localization of the B3LYP/6-31G(d,p) orbitals agree with chemical intuition; the orbital energies track oxidation and reduction potentials, and maintain functional group integrity; and HOMO–LUMO energy gap sums the oxidation and reduction potentials from electrochemical measurements using this functional [65]. The use of B3LYP for both geometry optimization and molecular orbital study would be incompatible because the bond energy of B3LYP/6-31G(d,p) and the association energy for loosely bound systems (such as systems self-assembled by electrostatics, π-π interactions, van der Waals, etc.) are consistently much greater than the experimental ones, possibly due to the large base set incompleteness error (BSIE) associated with the 6-31G(d,p) [68,69]. In the computation of compound **2**, long alkoxy groups were replaced with methoxy to facilitate calculations.

## 3. Results and Discussion

### 3.1. Electroreduction of Terthiophene-C_60_ Dyads

**Figure 3:**
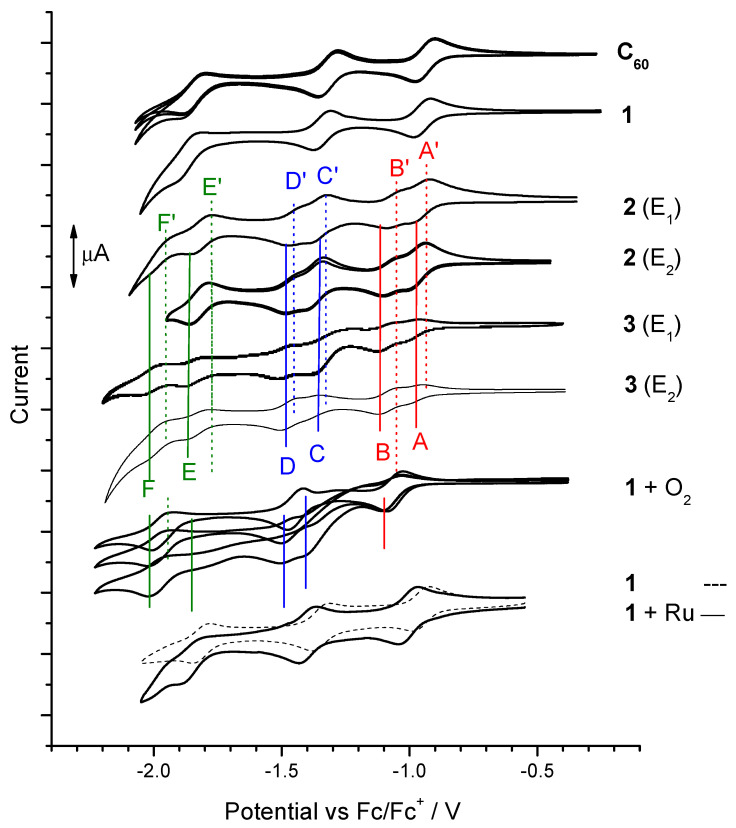
Voltammograms of reduction of **C_60_** and dyad **1–3** in electrolyte E_1_ or E_2_ in DCM, with (+O_2_) and without oxygenation in the rest of the cases, additionally in the presence of Ru_3_(CO)_12_. (+Ru).

The electroreduction of dyads **1**, **2,** and **3** and also **C_60_** was carried out in a solution of two different electrolytes: tetraethylammonium tetrafluoroborate (E_1_) and tetrabutylammonium tetrafluoroborate (E_2_) in dichloromethane. The electrochemical examination requires the presence of additional salt in the tested sample solution. An electrolyte is used as an ion conductor, but also to ensure that the tested polarized sample does not move away from the electrode. Admittedly, we have not found reports on the formation of fullerene complex with tetraethylammonium or tetrabutylammonium cations or any cations of standard electrolytes used in electrochemical study. Despite this, we thought that it was necessary to check the form of the complex with used electrolyte salts. Three reversible redox pairs were recorded with reduction peaks from the following reduced states of **C_60_**, with values of −0.98, −1.42, and −1.97 V for E_1_ electrolyte. At similar values, the reduction peaks of compound **1** were recorded: −1.05, −1.44, and −2.01 V. However, for dyads **2** and **3**, the CV courses for the reduction differed from the previous compounds, because in the same potential range not three but five and even six reduction peaks were observed with the values: −1.01; −1.10; −1.41; −1.50; −1.87 V (**2**) and −1.08; −1.18; −1.48; −1.55; −1.95; −2.01 V (**3**) in E_1_. There were no differences in the course or significant position of the peaks depending on the electrolyte used (E_1_ vs E_2_), and detailed data are presented in Table 1. Further analyses were performed only with the participation of the electrolyte E_1_ (Figure 3).

It is interesting that the six peaks appeared at once in the first CV run and especially already in the first stage of electroreduction in **2** and **3** solutions, so it was not the result of generating reactive species in the first reduction scan. While the three-step electroreduction via radical anion, dianion, and trianion states were recorded in **C_60_** and **1** solution sequentially, instead six-step electroreduction was present in solutions **2** and **3,** in the same potential range. It also should be noted that these CV runs were carried out in deoxygenated solutions to prevent reaction between oxygen and C_60_ radical. Additionally, six states of reduction were visible as well with and without the use of an ultrasonic bath (Figure 3).

**Figure 4:**
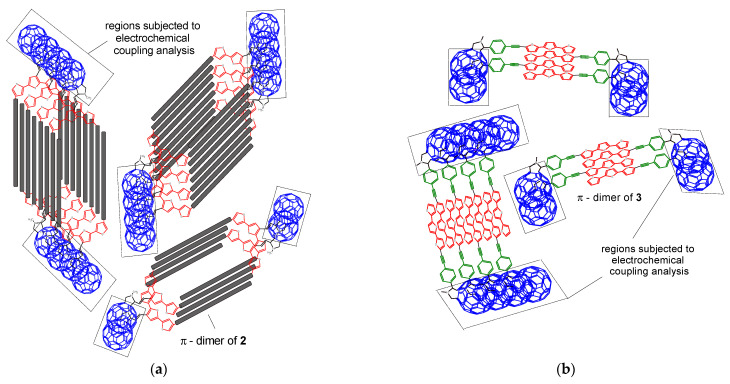
Structures of π-dimers and π-aggregates formed by **2** (**a**) and **3** (**b**).

Therefore, the following electroreduction scheme was proposed, with the possibility of spontaneous formation of dimers **(2)_2_** and **(3)_2_** in the solution (Figure 4). Interestingly, dimerization proceeds despite the fact that long substituents are present in the cation of electrolyte E_1_ and should rather prevent packing.

Thus, **2** and **3** dyads underwent six sequential, one-electron reductions of π-dimer (R-C_60_···C_60_-R), where two electrons were added successively to each of the two fullerene cages. We can distinguish here an individual reduction of each C_60_ cage in the dimer **(2)_2_** and **(3)_2_** giving the following A, B, C, D, and E reduction peaks as production: neutral—radical anion (Equation (1)), radicalanion—radical anion (Equation (2)), dianion—radical anion (Equation (3)), dianion—dianion (Equation (4)), dianion—trianion (Equation (5)), and trianion—trianion pair (Equation (6)). Electroreduction processes in a system of π-dimers of liquid crystalline C_60_ dyads (R-C_60_)-**2** and **3** with weakened forces of electrostatic repulsion go according to the following notation:

(1) $$A/A^{\prime }\;\;\;\;\;\;\;\;\;\;\;\;\;\left(R-C_{60}\right)\left(R-C_{60}\right)\overset{+1e^{-}}{\Leftrightarrow }{\left(R-C_{60}\right)}^{•-}\left(R-C_{60}\right)\;\;\;\;$$

(2) $$B/B^{\prime }\;\;\;\;\;\;\;\;{\left(R-C_{60}\right)}^{•-}\left(R-C_{60}\right)\overset{+1e^{-}}{\Leftrightarrow }{\left(R-C_{60}\right)}^{•-}{\left(R-C_{60}\right)}^{•-}\;\;\;\;\;\;\;$$

(3) $$C/C^{\prime }\;\;\;\;\;{\left(R-C_{60}\right)}^{•-}{\left(R-C_{60}\right)}^{•-}\overset{+1e^{-}}{\Leftrightarrow }{\left(R-C_{60}\right)}^{2-}{\left(R-C_{60}\right)}^{•-}\;\;\;\;\;$$

(4) $$D/D^{\prime }\;\;\;{\left(R-C_{60}\right)}^{2-}{\left(R-C_{60}\right)}^{•-}\overset{+1e^{-}}{\Leftrightarrow }{\left(R-C_{60}\right)}^{2-}{\left(R-C_{60}\right)}^{2-}\;\;\;\;\;\;$$

(5) $$E/E^{\prime }\;\;\;\;{\left(R-C_{60}\right)}^{2-}{\left(R-C_{60}\right)}^{2-}\overset{+1e^{-}}{\Leftrightarrow }{\left(R-C_{60}\right)}^{3-}{\left(R-C_{60}\right)}^{2-}\;\;\;\;\;\;$$

(6) $$F/F^{\prime }\;\;\;\;{\left(R-C_{60}\right)}^{3-}{\left(R-C_{60}\right)}^{2-}\overset{+1e^{-}}{\Leftrightarrow }{\left(R-C_{60}\right)}^{3-}{\left(R-C_{60}\right)}^{3-}\;\;\;\;\;$$

The proximity of the reduction potentials between A and B peaks suggests that the electrons were added first to one C_60_ cage and then to the second. Interestingly, π-dimers **(2)_2_** and **(3)_2_** can undergo sequential reversible reductions. The separation between the two components A-B, C-D, and E-F increased, which could result from the effects of increasing Coulombic repulsion [70] and/or variable interaction between electron spins [71]. There also seems to be a significant π-conjugation occurring between C_60_ cages in these dimers, which can be found in covalently bonded dimers e.g., C_120_ [72], C_30_ [73], and C_60_-O-C_60_ [74]. Such a dimer was produced from precursor **1**, which, although not dimerized in E_1_ and E_2_ spontaneously, dimerized in the presence of oxygen, but only after exceeding the first reduction peak, where oxygen could couple with C_60_ radical anion to C_60_-O-C_60_. Then, in the second state of reduction, we already observed two reduction peaks from the reduction of each C_60_ cage separately in C_60_-O-C_60_ to dianion–radicalanion and dianion–dianion (Figure 3: **1 + O_2_**). However, already the preparation of the dimer by coordination with the Ru ion did not cause separation of these reduction peaks, usually like in the case with covalently bonded dimers (Figure 3: **1 + Ru**). Thus, the postulated dimers **(2)_2_** and **(3)_2_** must have the character of a dimer with good π-electron communication, but also have the nature of non-covalent bonding due to their instability, which is indicated by the reversibility of each stage of the reduction process.

**Figure 5:**
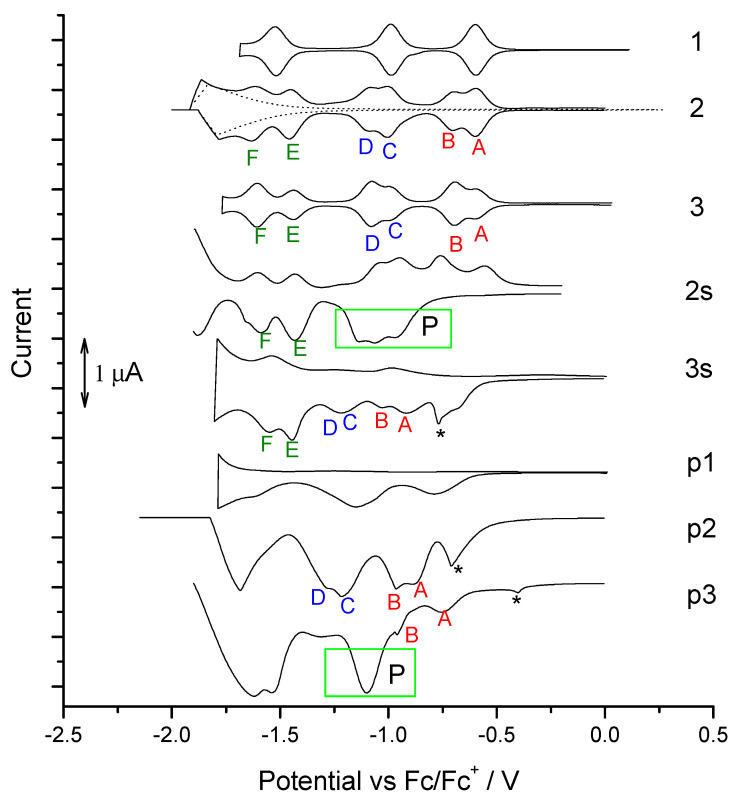
Differential voltammograms of reduction for: dyads **1–3** in solution in E_1_/DCM, in solid state (**s2**, **s3**) in E_1_/ACN and **p1**, **p2**, **p3**—their C_60_-pendant polymers with poly(terthiophene)’s scaffold in E_1_/DCM; *—pre-peak and following P peak derived from morphology rearrangement.

Next, electroreductions of **1**, **2,** and **3** were performed by differential voltammetry (Figure 5). The occurrence of a different area under the A relative to B and C relative to D, etc. was found, i.e., that is, in one-type reactions, which should proceed with the same number of electrons. Area disproportion of these paired peaks is the same for each successive reduction degree and seems to be characteristic giving the ratio of 2:1 or 1:2 for each following pair of A:B, C:D, and E:F for **2** or **3**, respectively. This phenomenon can be the result of both differences in electron tunneling effects between the two C_60_ cages in the dimer, and to the interactions of C60 with the donor of the dyad. In turn, comparing the area under the pairs of peaks A-B:C-D:E-F, we obtain the same area and thus the same charge (1:1:1).

Further, differential voltammetry measurements were also performed for compounds **2** and **3** in the solid state, denoted herein as **2s** and **3s**, deposited on a Pt electrode and polarized in propylene carbonate with an E_1_ electrolyte (Figure 5: **2s**, **3s**). Then, we observed the reduction peaks in the form of one wide “P” wave in the case of **2s**, which is probably related to the re-ordering process during the first full reductive polarization, but the six following peaks F’, E’, D’, E’, B’, and A’ were already observed in this returning half-cycle. It seems that only after the first full negative polarity, this system obtains a structure similar to that in the liquid. In turn, the spontaneous dimerization is effectively in the solid phase of **3** too, where the π-π interactions between terthiophene segments of **3s** are significant, so clearly peaks A, B, C, and D are visible during this electroreduction (Figure 5: **3s**).

### 3.2. Electroreduction of C_60-_Pendant Polymer with Poly(Terthiophene)’s Scaffold

Electrooxidation of **1**, **2,** and **3** was performed (Figure S1) and then the reduction processes of the product and corresponding precursor were compared (Figure 5: **p(1)**, **p(2)**, **p(3)**).

**Figure 6:**
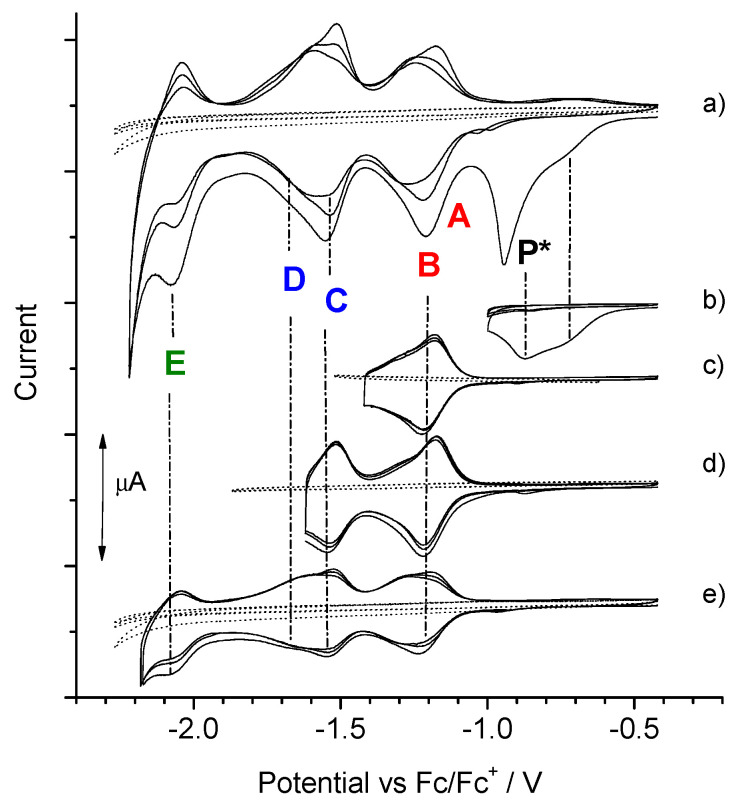
1st–3th voltammetric cycles of **p(2)** reduction in E_1_/DCM (**a**,**b**); 4th-6th voltammetric cycles (**c**–**e**); P*—pre-peak derived from morphology rearrangement; lower potential limited after: E peak (**a**,**e**); P* pre-peak (**b**), B peak (**c**) and C peak (**d**).

**Figure 7:**
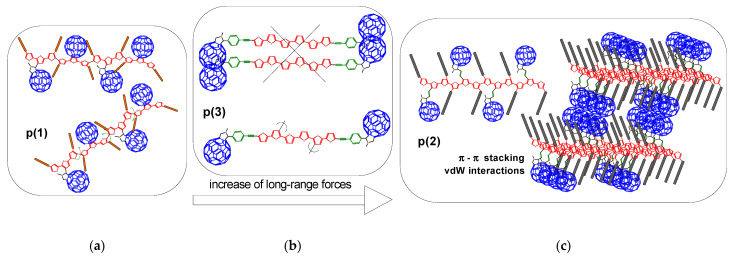
Structures of polymers **p(1)** (**a**), **p(3)** (**b**) and **p(2)** (**c**) with different extended of π-conjugation in donor unit (anodic electropolymerization) and π-aggregates formed in **p(2)**.

Oxidation of **1** and **2** produced pendant polymers **p(1) and p(2)**, respectively, with side C_60_ groups along the poly(terthiophene) chain, for which the electroreduction revealed A, B, C, and D peaks in the case of **p(2)** (Figure 5: **p(2)**). The pre-peak marked with the asterisk (*) comes from a physical process related to the rearrangement of the **p(2)** structure due to the removal of anions previously trapped in the anodic polymerization [75]. To confirm this, a reduction was also performed when the potential was inverted after the pre-peak (*) and next, each reduction state (Figure 6b–e). We observe here the irreversibility of the sharp pre-peak (*) and the reversibility of the redox couples A/A’; B/B‘, C/C’, and D/D’, while A/A’-B/B ‘and C/C’-D/D‘ are separated by a much smaller potential barrier than in a liquid. So it seems that the C_60_ cages are even organized in columns allowing for greater π-delocalization (Figure 7: **p(2)**). On the other hand, reduction CVs for **p(1)** are characteristic for isolated C_60_ cages, because short hexyl chains do not provide a higher degree of order in such polymer, where the chain separates the side groups from each other (Figure 7: **p(1)**).

It was initially assumed that the oxidized terthiophene fragment of **3** would dimerize via the α-position into the C_60_-linker-(hexathiophene)-linker-C_60_ triad. Such presence of hexathiophene segment in the product would contribute to increasing the length of the effective π-delocalization, π-π stacking and π-dimerization. However, the voltamperograms of the **3** oxidation process shows that there is a shift in the onset of the oxidation peak of the product **p(3)** in the subsequent cycles (Figure S1). The assumed coupling solely through α-positions of dimerizing terthiophene does not occur and probably β-positions are also active in dimerization or subsequent branching polymerization, where π-delocalization is broken in product **p(3)** (Figure 7: **p(3)**). This is also confirmed by the reduction differential voltammogram carried out for this product, where the clear reduction double peaks typical for π-paired C_60_ did not occur and could take place more efficiently if there was also stacking of the π-linear terthiophene chains (Figure 5: **p(3)**).

### 3.3. DFT Calculation

**Figure 8:**
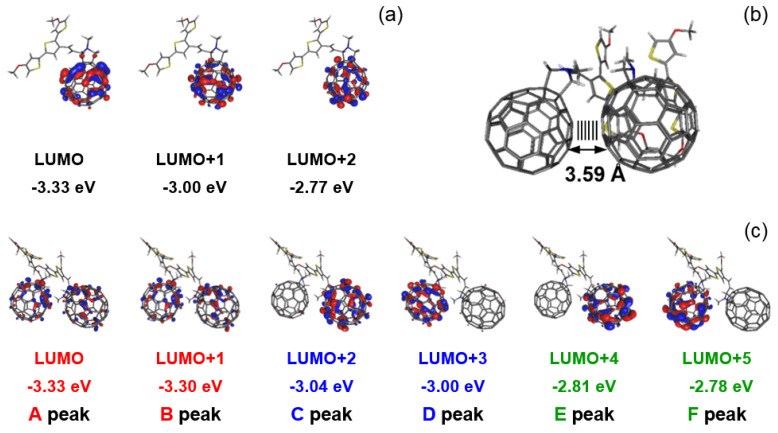
Shapes and energies of LUMO orbitals of **2** (**a**) and π-dimers **(2)_2_** (**c**) in neutral state, calculated at B3LYP/6-31G(d,p); optimized structure of this dimer with marked distances between C_60_ cages (**b**).

Geometry optimization was performed and presented for visualization and to give energy values of frontier orbitals for dyads **2** and **3**, and their dimers: **(2)_2_** and **(3)_2_** (Tables S1–S3-precursors; Tables S5 and S6-dimers). Spin densities of the reduced form of **2** and **3** are presented in Table S4. Optimization of dyad structures of **2** and **3** showed the presence of strong π-interactions in these dimer systems **(2)_2_** and **(3)_2_**, which may contribute to the reduction of the distance between the fullerene cages, resulting from interactions also between the alkoxy and or terthiophene units, which immobilizes the C_60_ cages to the distance 3.56 Å for both dimers (Table S7). For the isolated molecule **2** and **3**, the values of the following LUMO, LUMO + 1, and LUMO + 2 levels were obtained: −3.33; −2.99; −2.77 eV and −3.35; −3.01; −2.79 eV, respectively. Whereas for π-dimers, already six LUMO levels in the same energy range are visible: −3.33; −3.30; −3.04; −3.00; −2.81; and −2.78 eV for **(2)_2_** and −3.37; −3.37; −3.03; −3.02; −2.87; and −2.78 eV for **(3)_2_** (Figure 8). This means that instead of three reduction peaks registered for the monomer, six following steps could be registered during dimer reduction at the same potential window.

### 3.4. ESR Spectroscopy

**Figure 9:**
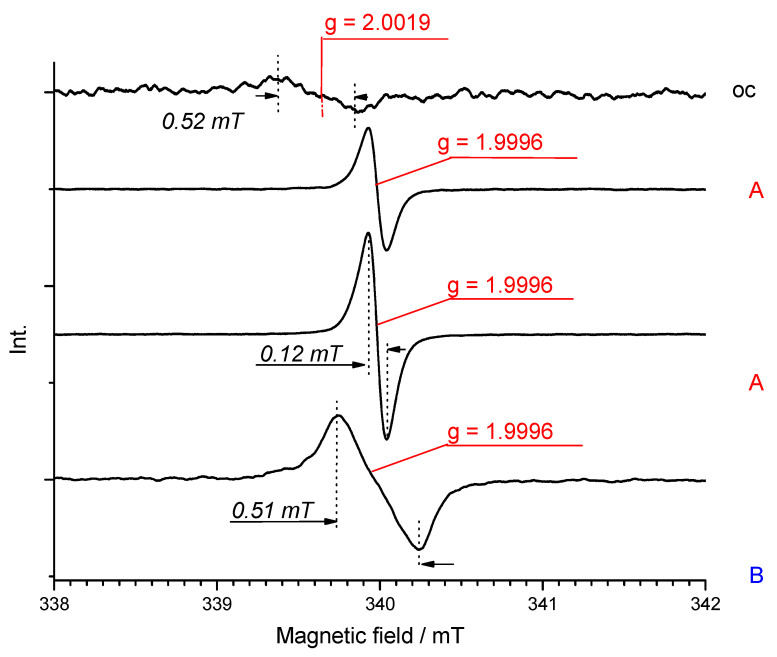
The change in ESR signal in **3** solution in E_1_/DCM, 0.6 mT (modulation width); 200 (amplitude).

A gradual electroreduction of **C_60_**, **1**, **2,** and **3** in the solution was performed. First, the states without potential applied (“oc”—open cell) were measured. ESR signal was observed only in cases of **1** (Figure S3) and **3** (Figure 9), which may be related to the presence of the radical cation–radical anion pair as an effect of the CT process. The presence of a suitable donor in the C_60_ dyad could have a significant impact through the charge transfer effect (CT) on the fullerene dimerization process in the solution. Fullerene complexes even with partial charge transfer can show dimerization [76]. Finally, the CT effect can also be the result of transfer from the dyad donor via the ligand (intramolecular CT) or without it (intra- or intermolecular CT) to the acceptor [77]. However, we did not observe CT effects in a dimerizing **2** solution, which could be easily demonstrated by ESR.

**Figure 10:**
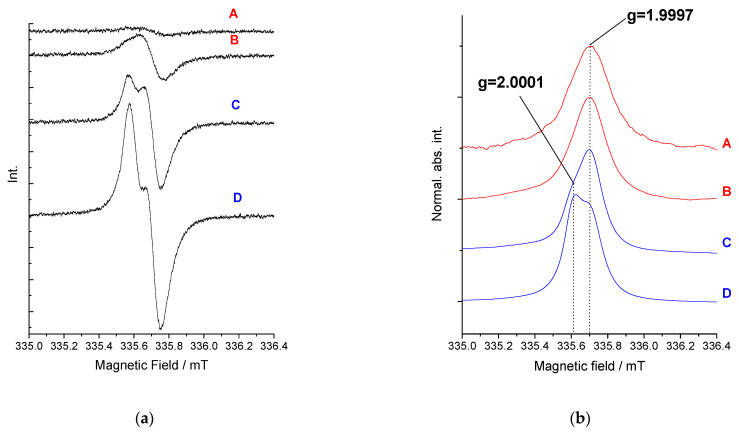
The change in ESR signal as intensity (**a**) and normalized absorption intensity (**b**) at A and B potential in **2** solution in E_1_/DCM, 0.6 mT (modulation width); 200 (amplitude).

Next, the sample solution was polarized at the peak of the first reduction stage (**C_60_**, **1** samples) and within the potential of the system of the first two reduction peaks A and B (**2**, **3** samples). ESR spectrum of radical anion of fullerene cage C_60_^•−^ consists of a doublet state manifested by a single sharp line at g-factor and a linewidth respectively: 1.9995, 0.27 mT (C_60_) (Figure S2); 1.9995, 0.28 mT (**1**) (Figure S3); 1.9997 (0.14 mT) (**2**) (Figure 10a,b); and 1.9996 (0.12 mT) (**3**) (Figure 9) [78]. This first recorded signal does not move, change its character, or decrease with successive reduction steps to the second reduction stage, which can be related to the reaction between dianion C_60_^2−^ with neutral particle to produce two radical anions. This first recorded signal does not shift or change its character and only increases with successive reduction steps to the second reduction stage, which can be related to the reaction between dianion C_60_^2−^ with neutral particles to produce two moieties of C_60_^•−^ radical anion, and was observed in the case of **C_60_** and **1** sample. The ESR spectra of radical anions of fullerene dyads have similar or slightly higher g values than those of pristine C_60_. The spectrum only differs with the further electroreduction of samples **2** and **3**. Electroreduction of **2** under the potential of C and D peaks reveals the appearance of a new peak next to that previously observed at g equal to 1.9997 (0.14 mT) (Figure 10b). For proper interpretation, it was necessary to integrate the spectra and then decompose them, which revealed two components, as shown in Figure S4. The novel peak is characterized by a g value equal to 2.0001 and width comparable to the first (0.12 mT). If this species acted as a dianion with independent C_60_ cages, a spectrum similar to these in case **C_60_** and **1** solution would be expected. In contrast, [C_60_-C_60_]^2−^ displays both singlet (g = 1.9997) and triplet ESR features with higher g values (2.0001) [79]. In particular, the appearance of the triplet state indicates the existence of a state with significant interaction between the two electrons, and the most obvious means of communication would come about between π-π overlap in the region where the two cages are held closest—in the liquid crystal phase as a triplet π-diradical dianion. It is an interesting observation because generally the stability of the C_120_ dimer is low and undergoes dissociation upon heating or in the following reduction [80]. Thus, the observed π-dimerization is not a result of the formation of an ionic complex between a dyad and cation, in addition CT transition is not required here; however it is the result of the presence of close-packed structures and reduction in the distance between C_60_ cages in liquid-crystalline fullerene dyads.

**Figure 11:**
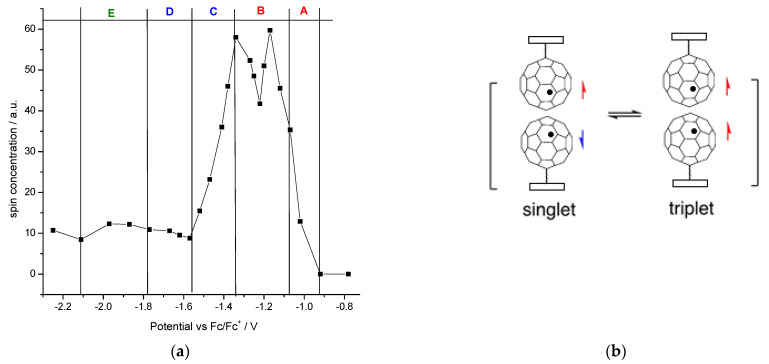
Spin concentration under **p(2)** electroreduction in E_1_/DCM (**a**); equilibrium shifting between the triplet and singlet states of radical anion pairs, manifesting as fluctuations in the spin concentration within peak B (**b**).

The changes of spin concentration should have been assessed due to the possibility of coupling fullerene radicals in a well-ordered polymer such as **p(2)** in order to transform the double-cable conjugated polymer from lower to better crystallization in both types of charge transfer domains, positive poly(terthiophene) and negative poly(fullerene) scaffold [81]. For this purpose, changes in the ESR spectrum during **p(2)** electroreduction were recorded. This measurement had the advantage over the measurement of the monomer solution in that all the polarized material remained on the electrode and here all the C_60_ as side groups were reduced at once. As a solution, polarized molecules can be reacted with neutral molecules as previously shown. The aim of the study was to find out whether the polymer could obtain ordered structures favoring the spins paring, as in the solution. A double integration of the ESR signal was carried out to determine the concentration of spin species in **p(2)**, and the variability of their concentration depending on the potential is shown in Figure 11a.

Through the gradual polarization of this system in the range of reduction peaks A and B, first, we observed a systematic increase in the concentration of spins and then a doubling of this concentration within the limits of peak B. However, we noticed some fluctuations in the concentration values of the potential of B, which may be related to the formation of a certain equilibrium between the triplet and singlet states of the pair of radical anions of two closely located C_60_ units (Figure 11b). Next, polarization at the potential of C peak was manifested by a sudden decrease in the concentration of spins, which indicates a silent dianion in the ESR technique, because shared covalent bonding of dianion fullerenes in the solid phase increased. Further reduction of the potential of D and E peaks kept the spin concentration at a similar level. However, a deeper analysis here is debatable due to the possibility of reacting a trianion of fullerene with dichloromethane. Electroreduction of **3** at the C-D peaks resulted in a spectrum widening to 0.51 mT. However, the signal from the singlet was still dominant at g 1.9996 V (Figure 10). Thus, decoupling between C_60_ cages was visible in the polymer as well, due to the reversible process of transition between singlet and triplet states.

## 4. Conclusions

The main objective of this work was to study the specific ordering of fullerene dyads as monomers in solutions, and the ability of such precursors to dimerize in liquid-crystalline solutions. The nature and strength of intermolecular interactions are crucial in many aspects of research, beginning with the physical properties (solubility, mechanical, electrical properties, etc.) and ending with the reactivity of molecules and biochemical applications. In the presented research, we showed that low-range interactions make it possible to “immobilize” C_60_ cages in the liquid crystal phase and their significant electronic interactions. Such ordering makes it possible to control and regulate the electrically conductive properties of low molecular weight semiconductors, and liquid crystalline dyads have now become interesting as precursors for ambipolar polymers obtained after both anodic or anodic-cathodic polarization. Further, electrochemical and spectroelectrochemical studies allow for effective recognition of such difficult-to-observe interactions in nature such as π-dimerization phenomena, which has not been successful with many techniques.

## Figures and Tables

**Table 1:** Summary of the potential (V) of cathodic/anodic pairs of samples calculated versus Fc/Fc+ couple from the CV in E_1_ and E_2_ electrolyte in DCM.

	I/I’		II/II’		III/III’	
**C_60_**/E_1_	−0.98/−0.91		−1.42/−1.31		−1.97/−1.89	
**1**/E_1_	−1.05/−0.96		−1.44/−1.36		−2.01/−1.90	
	**A/A’**	**B/B’**	**C/C’**	**D/D’**	**E/E’**	**F/F’**
**2**/E_1_	−1.01/−0.95	−1.10/−1.03	−1.41/−1.36	−1.50/−1.48	−1.87/−1.81	---
**2**/E_2_	−1.03/−0.96	−1.12/−1.08	−1.41/−1.34	−1.49/−1.46/	−1.85/−1.79	---
**3**/E_1_	−1.08/−1.00	−1.18/−1.12	−1.48/−1.40	−1.55/−1.48/	−1.95/−1.89	−2.01/−1.96
**3**/E_2_	−1.10/−0.96	−1.20/−1.16	−1.45/−1.36	−1.57/−1.48/	−1.93/−1.86	−2.05/−1.99

**A**, **A’**, **B**, **B’**, **C**, **C’**, **D**, **D’**, **E**, **E’**, **F** and **F’** correspond to those mention in Figure 3.

## Data Availability

The data presented in this study are available on request from the corresponding author.

## Supplementary Materials

The following are available online at www.mdpi.com/article/10.3390/polym13162816/s1, Figure S1. Voltammograms of oxidative electropolymerization of **1**, **2** and **3** in precursor solution (1 mM) in E_1_/dichloromethane (0.1 M) to produce polymers **p(1)**, **p(2)** and **p(3)** (left) and images of these polymers surfaces obtained using an optical Raman microscope under laser excitation of 514 nm (right). Figure S2. The change in ESR signal at I (first), II (second) and third (III) reduction peak in **C_60_** solution in E_1_/DCM, 0.6 mT (modulation width); 200 (amplitude) (oc–open cell, without polarization). Figure S3. The change in ESR signal at I (first), II (second) and third (III) reduction peak in **1** solution in E_1_/DCM, 0.6 mT (modulation width); 200 (amplitude) (oc–open cell, without polarization). Figure S4. Graphical method showing the decomposition of absorption ESR signal into two components under potential of B peak in **2** solution in E_1_/DCM, 0.6 mT (modulation width); 200 (amplitude). Table S1. Shape of frontier orbitals of 2 calculated at B3LYP/6-31G(d,p). The isovalue is equal to 0.03 e^−^/au^3^ in each case. Table S2. Shape of frontier orbitals of **2** (without alkoxy group) calculated at B3LYP/6-31G(d,p). The isovalue is equal to 0.03 e^−^/au^3^ in each case. Table S3. Shape of frontier orbitals of 3 calculated at B3LYP/6-31G(d,p). The isovalue is equal to 0.03 e^−^/au^3^ in each case. Table S4. Spin density of reduced form of monomers calculated at B3LYP/6-31G(d), isovalue is equal to 0.005 e^−^/au^3^.Table S5. Shape of frontier orbitals of **(2)_2_** dimer in neutral state, calculated at B3LYP/6-31G(d,p). The isovalue is equal to 0.03 e^−^/au^3^ in each case. Table S6. Shape of frontier orbitals of **(3)_2_** dimer in neutral state, calculated at B3LYP/6-31G(d,p). The isovalue is equal to 0.03 e^−^/au^3^ in each case. Table S7. Optimized structures of **(2)_2_** and **(3)_2_** dimers in neutal state, calculated at ϖB97X-D functional combined with 6-31G(d,p) basis set.
