# Supplementary material for: Exohedral Functionalization of Fullerene by Substituents Controlling of Molecular Organization for Spontaneous C60 Dimerization in Liquid Crystal Solutions and in a Bulk Controlled by a Potential
Source: Polymers (Basel). 2021 Aug 22;13(16):2816. doi: 10.3390/polym13162816 (PMC8401323; doi:10.3390/polym13162816)
Supplement: Supplementary file 1 [file polymers-13-02816-s001.zip › polymers-1329643-supplementary.pdf]

Exohedral functionalization of fullerene by substituents controlling of molecular organization for spontaneous C_{60} dimerization in liquid crystal solutions and in a bulk controlled by a potential

Malgorzata Czichy [1,*], Alessia Colombo [2], Pawel Wagner [3], Patryk Janasik [1], Claudia Dragonetti [2], Rathinam Raja [4], David L. Officer [3] and Leeyih Wang [4,5]

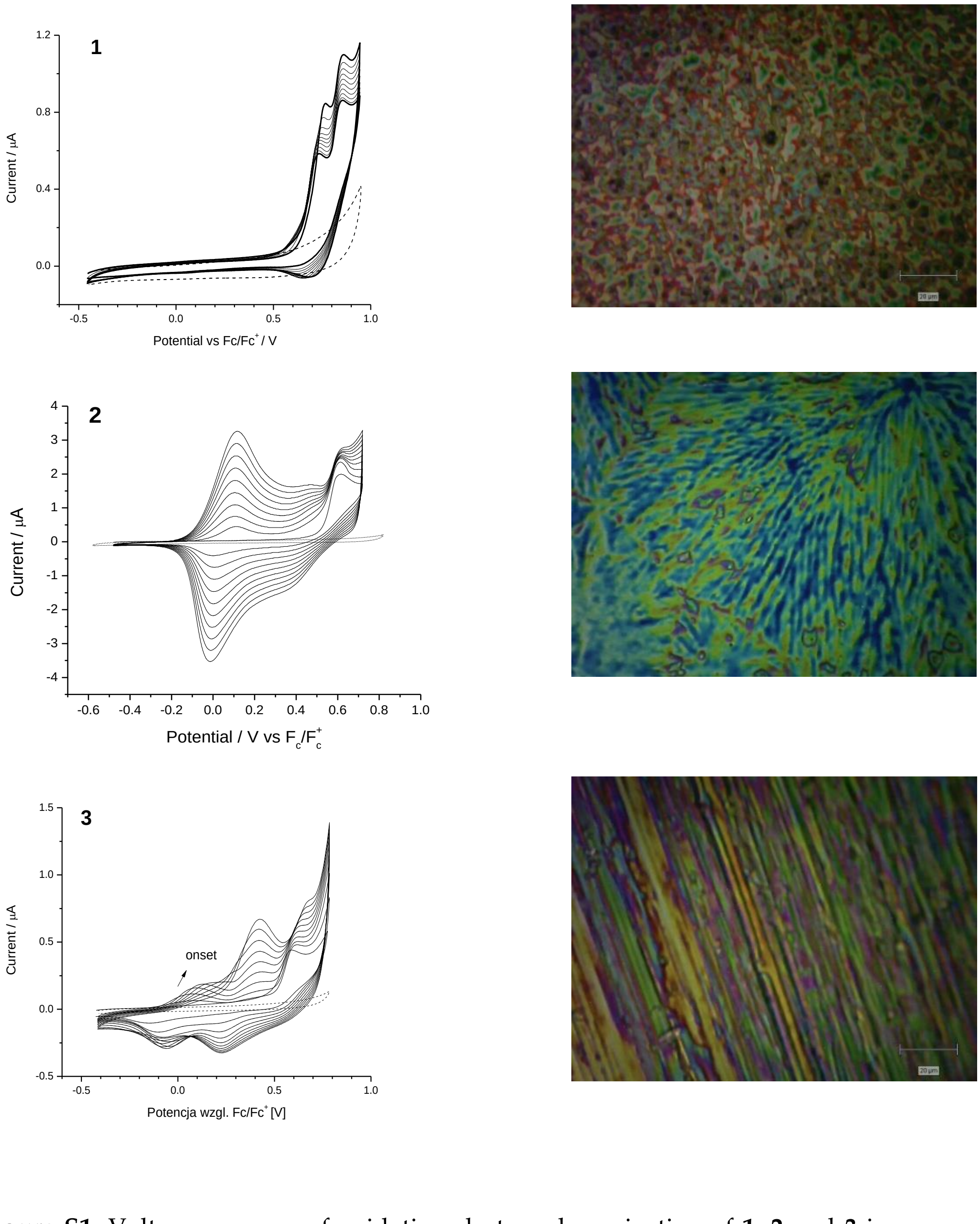

Figure S1. Voltammograms of oxidative electropolymerization of **1**, **2** and **3** in precursor solution (1 mM) in E1/dichloromethane (0.1 M) to produce polymers **p(1)**, **p(2)** and **p(3)** (left) and images of these polymers surfaces obtained using an optical Raman microscope under laser excitation of 514 nm (right).

Table S1. Shape of frontier orbitals of **2** calculated at B3LYP/6-31G(d,p).
The isovalue is equal to 0.03 e^-/au^3 in each case.

HOMO-1	HOMO	LUMO
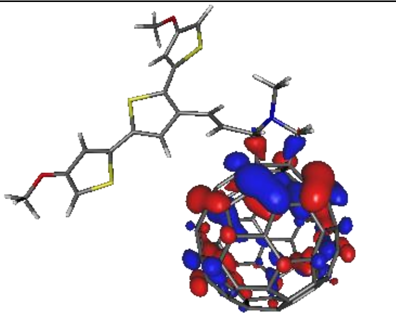	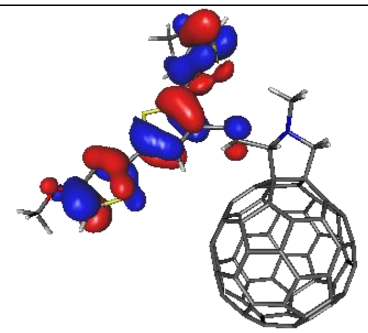	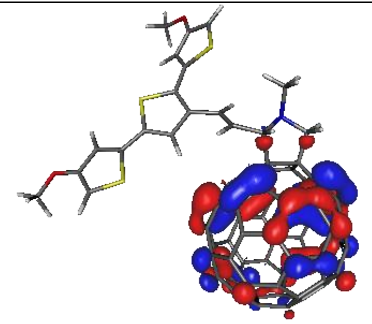
**-5.32**	**-5.15**	**-3.33**
**LUMO+1**	**LUMO+2**	**LUMO+3**
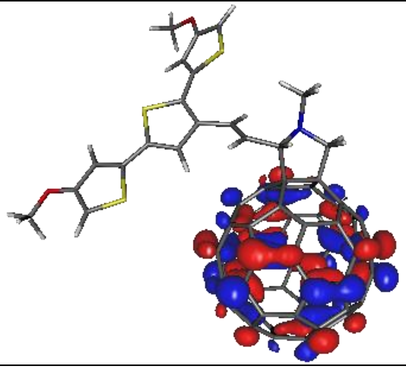	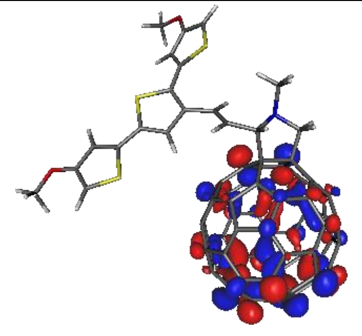	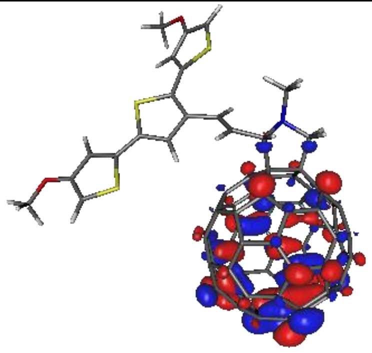
**-2.99**	**-2.77**	**-2.20**
**LUMO+4**	**LUMO+5**	**LUMO+6**
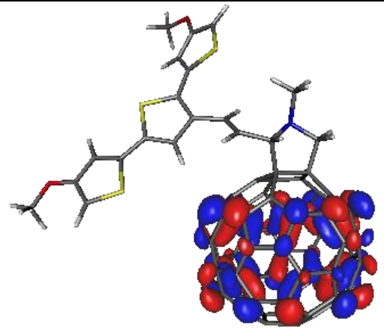	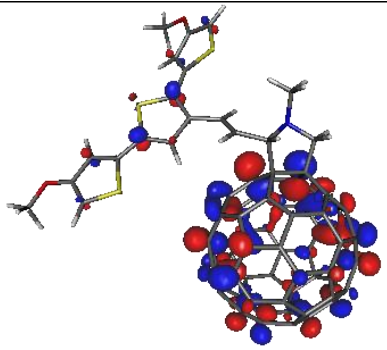	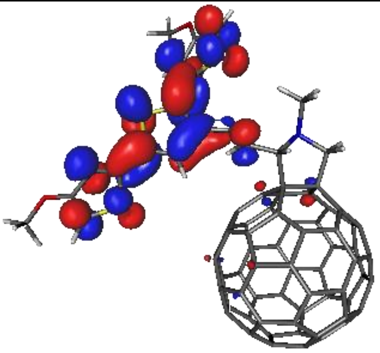
**-1.81**	**-1.59**	**-1.56**

Table S2. Shape of frontier orbitals of **2 (without alkoxy group)** calculated at B3LYP/6-31G(d,p). The isovalue is equal to 0.03 e^-/au^3 in each case.

**HOMO-1**	**HOMO**	**LUMO**
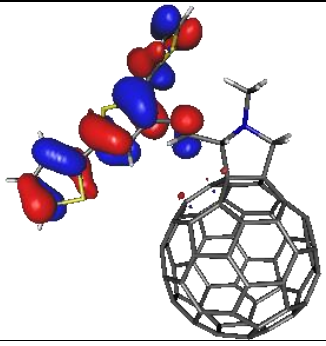	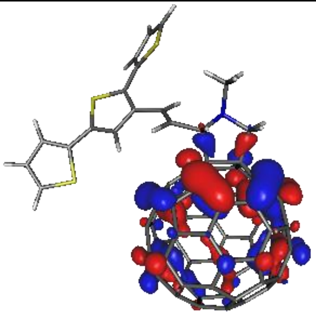	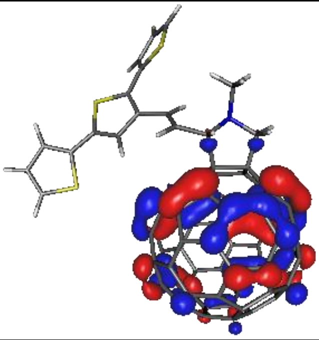
**-5.38**	**-5.32**	**-3.33**
**LUMO+1**	**LUMO+2**	**LUMO+3**
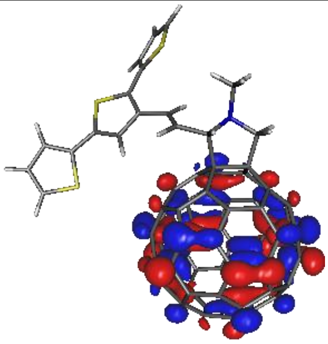	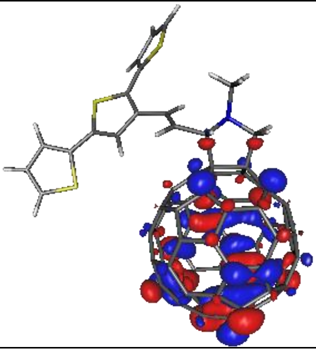	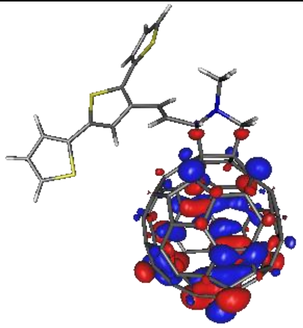
**-3.00**	**-2.77**	**-2.20**
**LUMO+4**	**LUMO+5**	**LUMO+6**
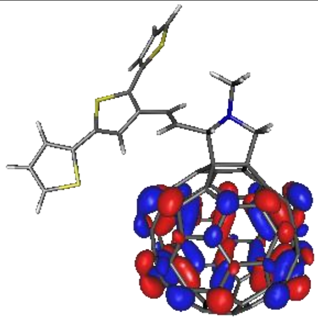	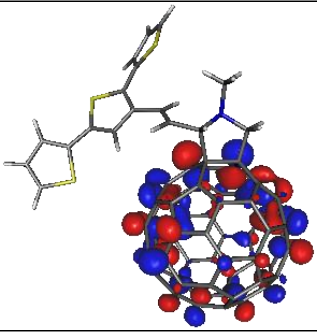	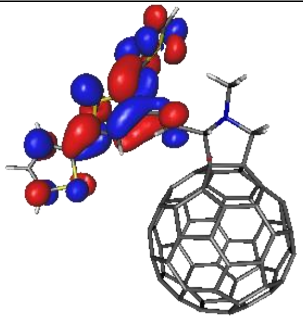
**-1.81**	**-1.59**	**-1.50**

Table S3. Shape of frontier orbitals of **3** calculated at B3LYP/6-31G(d,p).
The isovalue is equal to 0.03 e^-/au^3 in each case.

HOMO-1	HOMO	LUMO
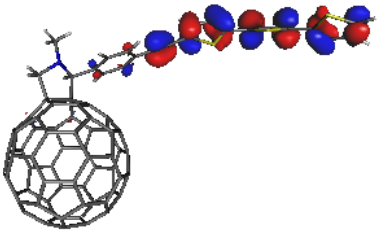	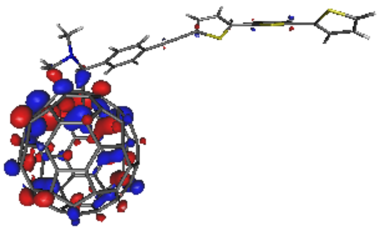	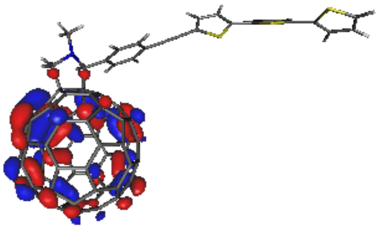
-5.36	-5.34	-3.35
LUMO+1	LUMO+2	LUMO+3
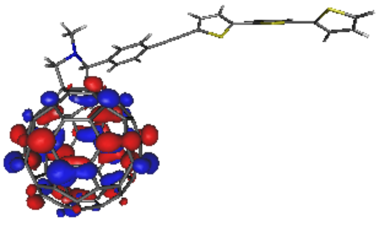	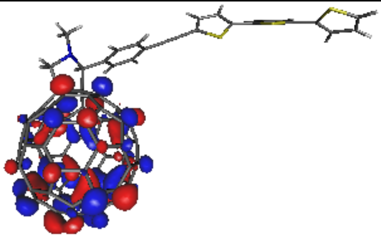	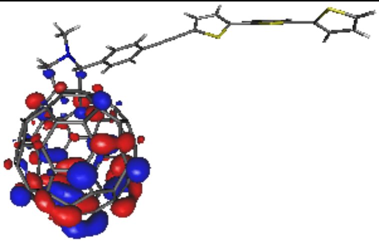
-3.01	-2.79	-2.22
LUMO+4	LUMO+5	LUMO+6
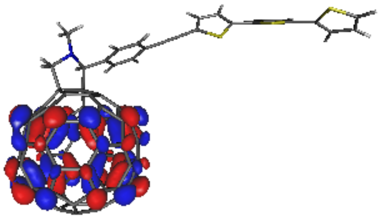	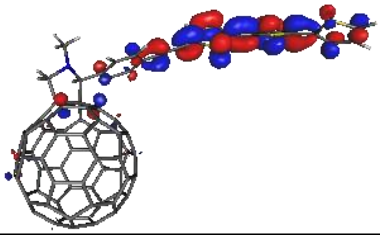	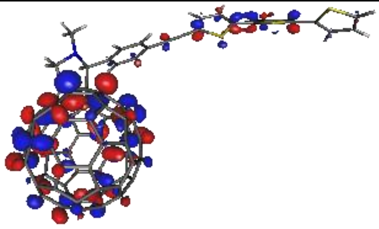
-1.82	-1.63	-1.61

Table S4. Spin density of reduced form of monomers calculated at B3LYP/6-31G(d), isovalue is equal to 0.005 e-/au^3.

2	3
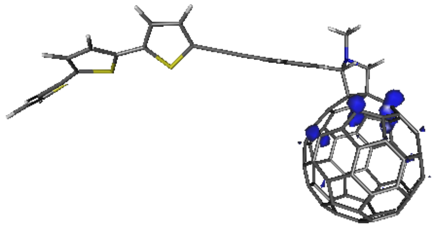	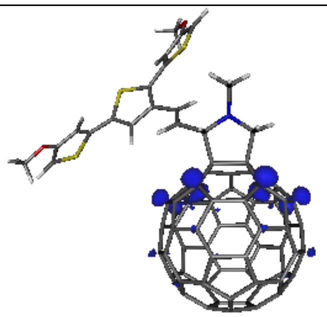
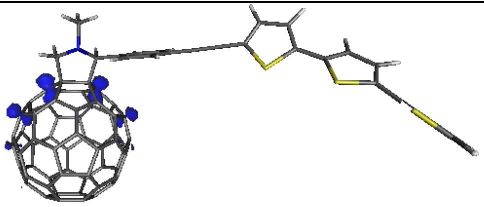	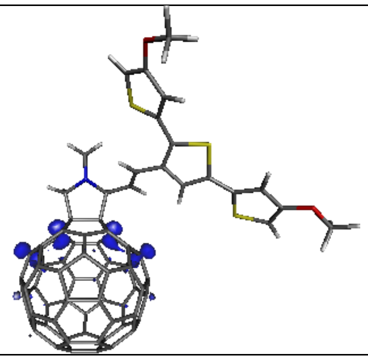
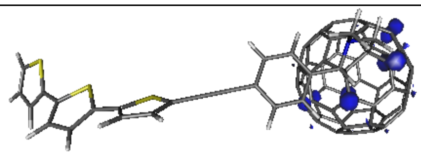	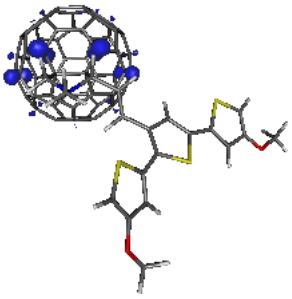

Table S5. Shape of frontier orbitals of **(2)**$_2$ dimer in neutral state, calculated at B3LYP/6-31G(d,p). The isovalue is equal to 0.03 e$^-$/au^3 in each case.

**HOMO-3**	**HOMO-2**	**HOMO-1**
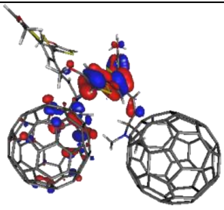	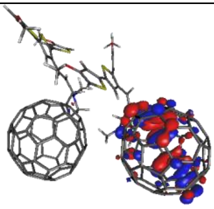	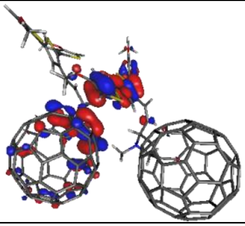
**-5.37**	**-5.31**	**-5.24**
**HOMO**	**LUMO**	**LUMO+1**
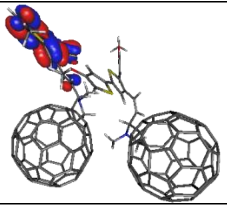	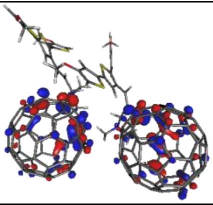	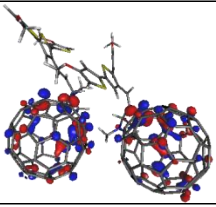
**-4.99**	**-3.33**	**-3.30**
**LUMO+2**	**LUMO+3**	**LUMO+4**
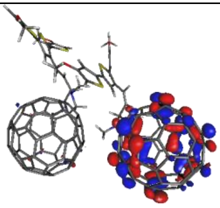	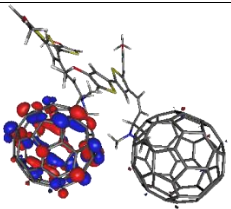	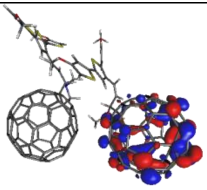
**-3.04**	**-3.00**	**-2.81**
**LUMO+5**	**LUMO+6**	**LUMO+7**
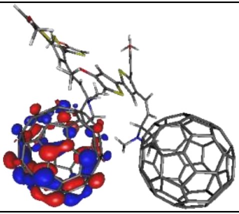	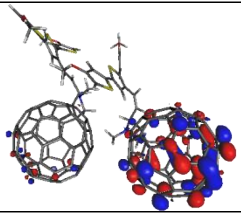	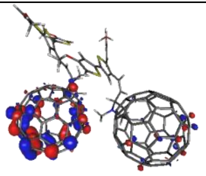
**-2.78**	**-2.21**	**-2.19**
**LUMO+8**	**LUMO+9**	**LUMO+10**
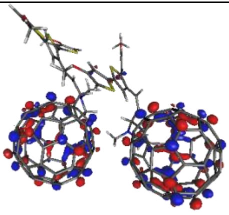	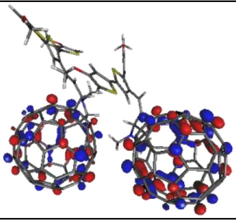	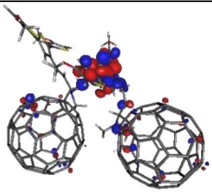
**-1.84**	**-1.81**	**-1.73**
**LUMO+11**		
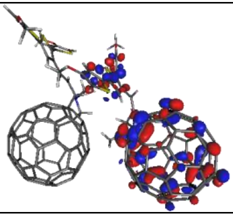		
**-1.62**		

Table S6. Shape of frontier orbitals of **(3)**$_2$ dimer in neutral state, calculated at B3LYP/6-31G(d,p). The isovalue is equal to 0.03 e$^-$/au^3 in each case.

**HOMO-3**	**HOMO-2**	**HOMO-1**
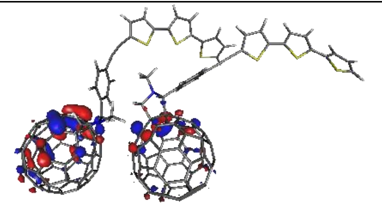	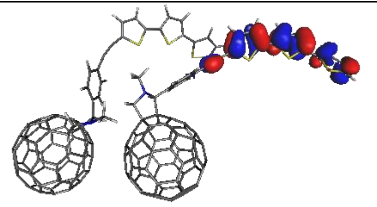	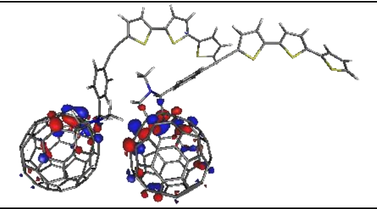
**-5.35**	**-5.31**	**-5.30**
**HOMO**	**LUMO**	**LUMO+1**
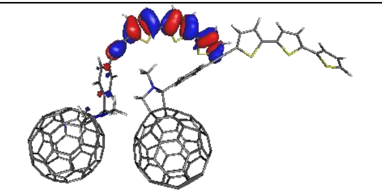	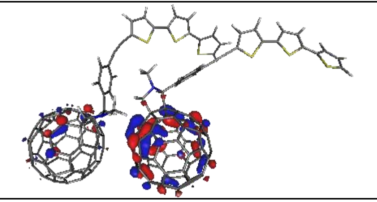	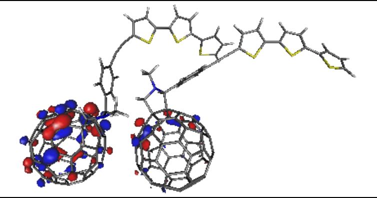
**-5.26**	**-3.37**	**-3.37**
**LUMO+2**	**LUMO+3**	**LUMO+4**
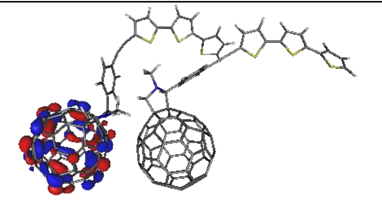	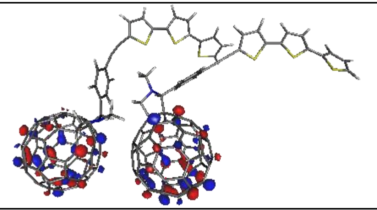	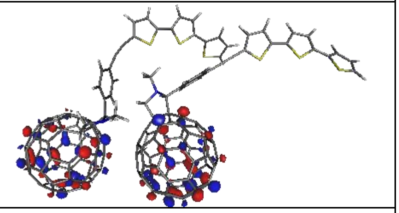
**-3.03**	**-3.02**	**-2.84**
**LUMO+5**	**LUMO+6**	**LUMO+7**
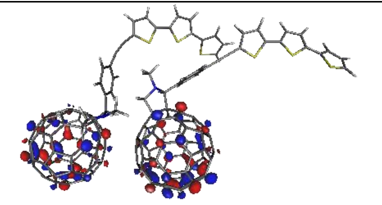	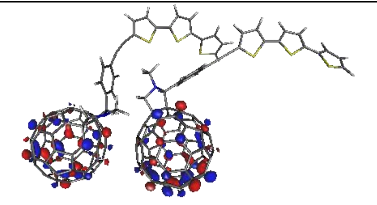	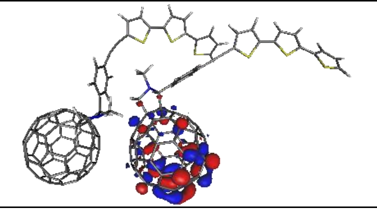
**-2.78**	**-2.24**	**-2.23**
**LUMO+8**	**LUMO+9**	**LUMO+10**
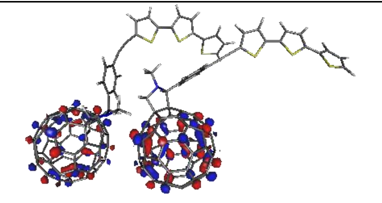	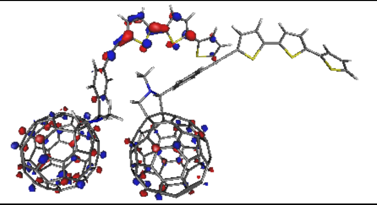	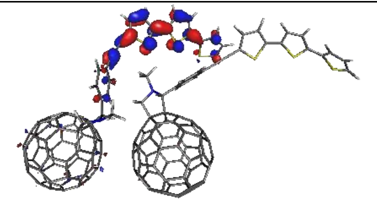
**-1.85**	**-1.83**	**-1.82**
**LUMO+11**		
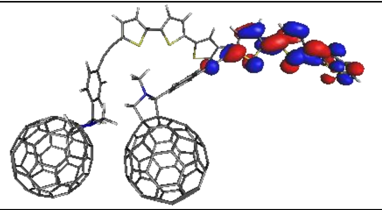		
**-1.73**		

Table S7. Optimized structures of **(2)**2 and **(3)**2 dimers in neutal state, calculated at ϖB97X-D functional combined with 6-31G(d,p) basis set.

**(3)2**	**(2)2**
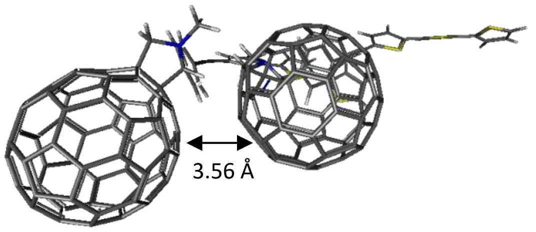	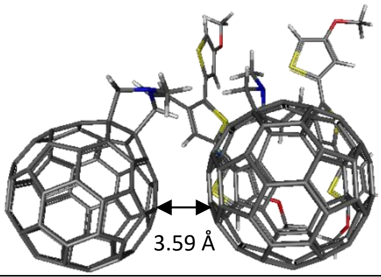
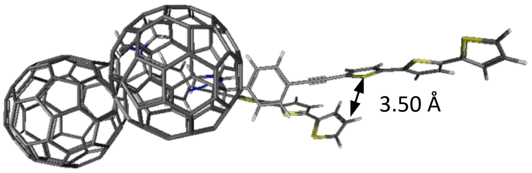	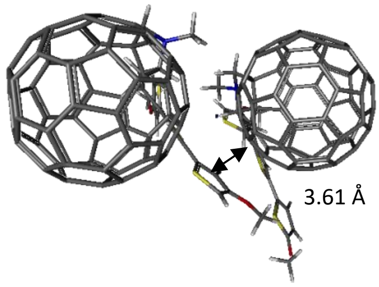

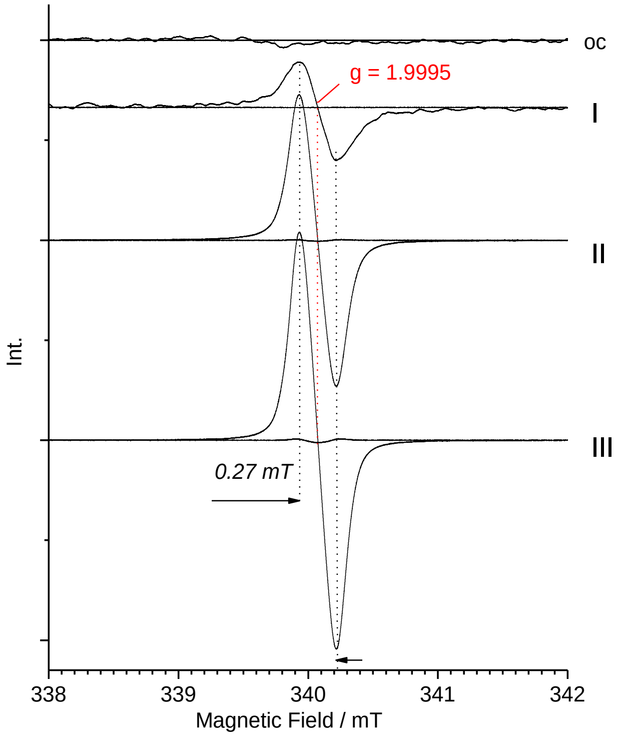

Figure S2. The change in ESR signal at I (first), II (second) and third (III) reduction peak in **C_{60}** solution in E1/DCM, 0.6 mT (modulation width); 200 (amplitude) (oc – open cell, without polarization).

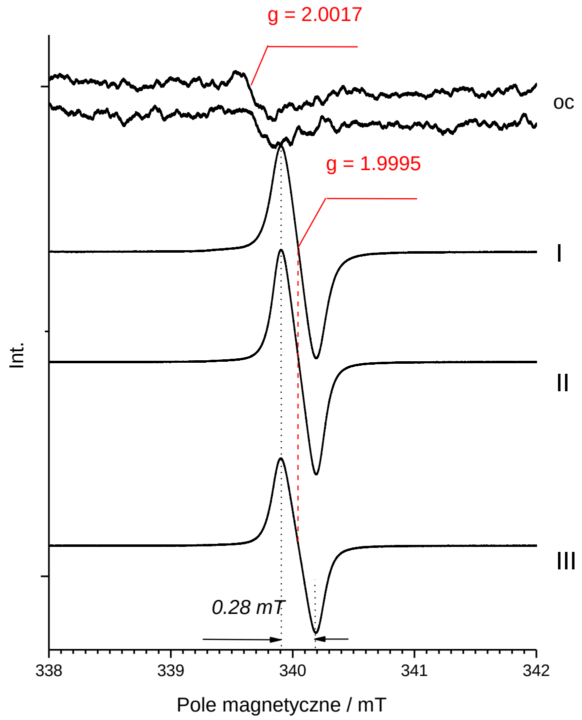

Figure S3. The change in ESR signal at I (first), II (second) and third (III) reduction peak in **1** solution in E1/DCM, 0.6 mT (modulation width); 200 (amplitude) (oc – open cell, without polarization).

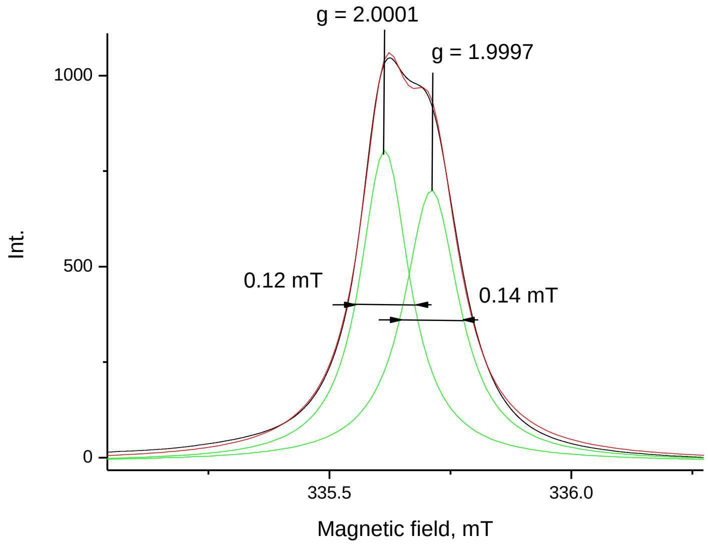

Figure S4. Graphical method showing the decomposition of absorption ESR signal into two components under potential of B peak in **2** solution in E1/DCM, 0.6 mT (modulation width); 200 (amplitude).
